# Effect of single-dose low-level helium-neon laser irradiation on orthodontic pain: a split-mouth single-blind placebo-controlled randomized clinical trial

**DOI:** 10.1186/s40510-015-0102-0

**Published:** 2015-09-29

**Authors:** Farhad Sobouti, Maziar Khatami, Nasim Chiniforush, Vahid Rakhshan, Mahsa Shariati

**Affiliations:** Department of Orthodontics, Dental Faculty, Mazandaran University of Medical Sciences, Sari, Iran; Department of Periodontics, Dental Faculty, Mazandaran University of Medical Sciences, Sari, Iran; Laser Research Center of Dentistry, Dental Research Institute, Tehran University of Medical Sciences, Tehran, Iran; Iranian Tissue Bank and Research Center, Tehran University of Medical Sciences, Tehran, Iran; Department of Dental Anatomy and Morphology, Dental Branch, Islamic Azad University, Tehran, Iran; Craniomaxillofacial Surgery Research Center, Shariati Hospital, Tehran University of Medical Sciences, Postal Code: 14174, North Kargar Ave., 16 Azar ST, Tehran, Iran

**Keywords:** Low-level laser therapy (LLLT), Helium-neon (He-Ne) laser, Orthodontic pain, Canine retraction, Placebo, Visual analog scale (VAS), Laser irradiation

## Abstract

**Background:**

Pain is the most common complication of orthodontic treatment. Low-level laser therapy (LLLT) has been suggested as a new analgesic treatment free of the adverse effects of analgesic medications. However, it is not studied thoroughly, and the available studies are quite controversial. Moreover, helium neon (He-Ne) laser has not been assessed before.

**Methods:**

This split-mouth placebo-controlled randomized clinical trial was performed on 16 male and 14 female orthodontic patients requiring bilateral upper canine retraction. The study was performed at a private clinic in Sari, Iran, in 2014. It was single blind: patients, orthodontist, and personnel were blinded of the allocations, but the laser operator (periodontist) was not blinded. Once canine retractor was activated, a randomly selected maxillary quarter received a single dose of He-Ne laser irradiation (632.8 nm, 10 mw, 6 j/cm^2^ density). The other quarter served as the placebo side, treated by the same device but powered off. In the first, second, fourth, and seventh days, blinded patients rated their pain sensed on each side at home using visual analog scale (VAS) questionnaires. There was no harm identified during or after the study. Pain changes were analyzed using two- and one-way repeated-measures ANOVA, Bonferroni, and *t*-test (*α* = 0.01, *β* > 0.99). This trial was not registered. It was self-funded by the authors.

**Results:**

Sixteen males and 11 females remained in the study (aged 12–21). Average pain scores sensed in all 4 intervals on control and laser sides were 4.06 ± 2.85 and 2.35 ± 1.77, respectively (*t*-test *P* < 0.0001). One-way ANOVA showed significant pain declines over time, in each group (*P* < 0.0001). Two-way ANOVA showed significant effects for LLLT (*P* < 0.0001) and time (*P* = <0.0001).

**Conclusions:**

Single-dose He-Ne laser therapy might reduce orthodontic pain caused by retracting maxillary canines.

## Background

The most common sequela of orthodontic treatment and one of its most significant problems is pain and discomfort [1–8]. Its intensity might be comparable with the highest ranked general pains such as wasp sting or spraining one’s ankle [1]. About 90 % of orthodontic patients find that orthodontic treatment is painful [9, 10]. Therefore, it is a critical deterrent to orthodontic treatment and a common cause of treatment discontinuation [1, 5–9, 11–14]. Despite its substantial clinical value, orthodontic pain is broadly neglected and underestimated [1, 7, 9, 14].

Various methods have been proposed to relieve orthodontic pain. According to recent reviews, the most effective approach is the administration of non-steroidal anti-inflammatory drugs (NSAID) [7, 11, 15]. However, besides their adverse effects, these analgesics might disrupt the osteoclastic mechanisms responsible for tooth movement by inhibiting prostaglandins and thus reduce the efficacy of orthodontic treatment [7, 11, 15]. Moreover, over-the-counter NSAID doses might inhibit tooth movement while might not necessarily relieve pain [9, 16]. Other methods for pain control include vibratory stimulation, transcutaneous electrical nerve stimulation, and chewing gum or plastic wafers [7, 11, 15]. However, the clinical application of such alternatives has been limited due to scant evidence, unclear influence, and poor tolerance [15]. Moreover, masticating firm objects might cause pain and discomfort [16].

Owing to unique advantages in bio-stimulation, pain relief, therapeutic effects, and lack of adverse effects, low-level laser therapy (LLLT) has attracted increasing attention in recent years [7, 11, 15]. This method might be relatively safer than some traditional approaches [11]. The efficacy of LLLT in reducing orthodontic pain has been studied recently [17–23]. Three systematic reviews/meta-analyses have been published in 2013 [24], 2014 [25], and 2015 [15], summarizing the emerging literature. Each of them independently concluded that the evidence is still lacking and further randomized clinical trials are necessary. This was mainly because of the rather small number of studies, controversial results, and methodological issues in almost all of them [15].

An issue with the methods was that most studies evaluated pain invoked by local separator placement [15], which cannot simulate common orthodontic pain caused by real tooth movements. A few studies have induced a generalized orthodontic pain by activating archwires [18, 26]; nevertheless, this method disallows effective split-mouth designs with proper contrasts between the left/right sides of the mouth.

Evaluating subjective phenomena like pain is a challenge, since it varies considerably between patients and even between different times in a single patient [15]. The best approach for dealing with such situations is conducting a split-mouth design which eliminates both interindividual and intra-individual confounders and thus allows deriving stronger conclusions based on smaller samples [15]. A way to assess localized pain (which is more reliable) in a split-mouth setup is to evaluate the pain caused by canine retraction. However, due to the design difficulties, only three studies have evaluated the pain of canine retraction [27–29], on 12 [29], 20 [27], and 30 patients [28].

Furthermore, all previous studies have evaluated aluminum-gallium lasers. There is no study on helium-neon (He-Ne) lasers. Therefore, we aimed to conduct this split-mouth clinical trial on the analgesic effect of a single-dose He-Ne laser irradiation on pain caused by canine retraction. The null hypotheses were the absence of any differences between the pains felt at laser or placebo sides as well as the absence of any changes in pain levels over time.

## Methods

This single-blind split-mouth placebo-controlled randomized clinical trial was performed (in 2014, Sari, Iran) on 60 bilateral maxillary canines retracted in 30 orthodontic patients (16 males, 14 females).

### Ethical considerations and potential harms

The ethics were approved by the university’s research committee, in accordance with the Helsinki declaration. This trial was not registered. Subjects or their parents were thoroughly briefed written and orally. Subjects could leave the study at their wish in any stage. They signed written consent forms.

The patients and the operator wore protective goggles. No harms were identified during the study, except for those being a routine part of the process of canine retraction (pain and discomfort).

### Screening for potential subjects

The patients were selected from attendees to a private orthodontic clinic in Sari, during 2013. The subjects were sequentially acquired until reaching the predetermined sample size.

### Eligibility criteria and sample

The inclusion criteria comprised the subjects’ willingness to participate, the indication for bilateral canine retraction (through the extraction of maxillary first premolars), the absence of any systemic diseases or mental disorders (e.g., anxiety disorders etc.), any history of medication intake as of 4 days before the treatment, any local or systemic condition affecting or inducing pain, as well as no history of previous orthodontic treatment of any kind. Patients were excluded if they did not return the completed questionnaires, used any analgesics during the trial period, were not available at the scheduled phone call, and if the treatment was interrupted [23, 30–32].

### Randomization

In this split-mouth design, each patient had a treatment side (real laser therapy) and a placebo side (simulated laser therapy) simultaneously. These sides were randomly pre-assigned in each patient, based on a random number table, by a periodontist who was the only person knowing the allocations (and did all the laser irradiations).

### Blinding

The patients, orthodontist, and personnel were blinded of the allocations. The results were coded. During the irradiation, personnel would leave the room, so only the periodontist would know the allocations (hence, single blind). Patients were not told of the experimental side. The placebo was the simulation of irradiation with the same duration but with the device turned off. Therefore, patients could not distinguish the placebo/experimental sides. Since the data were coded, the statistician did not know the grouping as well.

### Uniform treatment protocols

Orthodontic treatment plan included extraction of upper premolars for crowding correction or treatment of maxillary dental protrusion. Patients were treated using metal pre-adjusted brackets of slot 0.022 in. (MBT 3 M, Unitek, Monrovia, CA). After banding and bracket bonding, the stages of aligning and leveling were started. According to common treatment sequence, this treatment stage was done by nickel titanium archwires (Ormco, CA, USA) with diameters of 0.014, 0.016, and 0.018 in.

After finishing the aligning and leveling stages, canine retraction began using 0.018-in-stainless steel wires containing offset for canines, molar toe in and tip back in the mesial side of first molars. For more anchorage preservation, second molars were banded and engaged in wires in both sides. A closed power chain (3 M Unitek, USA) was used to apply forces of 150–175 g. Both sides were treated in the same session and immediately after each other. The side to begin the canine retraction with (left or right) was selected randomly as stated above. This randomization was absolutely independent of the randomization of the laser treatment side (left/right) and its order (being performed first or second). The force was standardized between both sides and among all patients, using a force gauge. All the canine retraction (and laser irradiation) procedures were performed at evening sessions (between 17 and 20 o’clock).

### Laser irradiation

All the experiments were performed in a single location and in the evening. In the experimental side, laser irradiation was conducted as follows: A single dose of laser emission was applied immediately after the initiation of force exertion. The used laser was He-Ne of red color (632.8 nm) emitted at a 10-mW power and an energy density of 6 J/cm^2^. The tip diameter was 5 mm. From the tooth CEJ to the end of the root apex, irradiation was separately done from the buccal and palatal. During the irradiation, the tip was directed perpendicular to the long axis of the tooth. Since the thickness of alveolar bone is greater over the apical part of the root, the duration of irradiation was decided to be as twice longer in the apical one half of the root, compared to its coronal half. Therefore, radical apical and coronal halves were irradiated for 40 and 80 s, respectively (on each of buccal or lingual sides). The phototherapy of each root section (buccal/lingual in combination with coronal/apical) was performed by a slow up-and-down movement of the device tip in a gentle touch with soft tissue, within the predetermined duration. The amount of laser irradiated at each point was standardized by the constant speed of the device tip being moved on the desired root section/side.

In the placebo side, the phototherapy was simulated [pretended] in terms of timing and every procedural detail with the same equipment, however, turned off. The patient was unaware of the placebo and experimental sides as well as the order of performing laser/placebo treatments.

### Pain measurement

In each patient, the pain was assessed on each side of the mouth using a visual analog scale (VAS). The patients were thoroughly instructed regarding filling VAS for left and right sides. A written instruction was as well given to them. The evaluations were done at home, on the first, second, fourth, and seventh days after imposing the force. Patients were called on their landline and/or mobile phones after 24, 48, 96, and 168 h after the treatment. On the phone, they were reminded of filling their VAS questionnaires.

The VAS was converted to 10 distances of equal length, between the 11 scores of 0–10. The score zero meant the absence of any pain/discomfort. The score 10 meant any pain considered intolerable by the patient OR causing the patient to seek emergency visits OR waking them from sleep [30].

### Statistical analysis

Descriptive statistics for pain levels, as the outcome, were calculated. The sample size was predetermined based on a pilot study of 17 patients, to obtain powers greater than 90 %. It sufficed to provide post hoc test powers greater than 99 % (*n* = 216 measurements, *α* = 0.01, mean difference = 1.213 ± 1.326). The difference between the control and experimental groups was assessed using a paired *t*-test of the SPSS program (v 20.0, IBM, USA). Repeated-measures one- and two-way analyses of variance (ANOVA) and a Bonferroni post hoc test were used to assess the effects of treatment and time on pain. The level of significance was set at 0.01.

## Results

More than 80 patients were assessed until 30 patients were enrolled. The excluded patients did not meet the inclusion criteria. Of the 30 included patients, 3 girls were dropped out of the study because of consuming analgesics or failure to answer the phone and fill the questionnaire on time. The remaining volunteers (16 males and 11 females) aged 12–21 years (mean = 15.3).

### Differences between pain sensed on placebo and laser sides

The average pain scores sensed in all 4 intervals on control and laser sides were 4.06 ± 2.85 and 2.35 ± 1.77, respectively. The paired *t*-test showed a significant difference between the pain level senses on each side (*P* < 0.0001). The paired *t*-test also detected significant differences between the treatment/placebo groups, at each of time intervals (Tables 1 and 2).

**Table 1:** Descriptive statistics for pain values

Day	Treatment	N	Mean	SD	CV	Min	Q1	Med	Q3	Max	95 % CI	
1	Placebo	27	6.63	1.94	29.3	2.0	5.0	7.0	8.0	10.0	5.86	7.40
	Laser	27	4.59	1.39	30.4	2.0	4.0	5.0	5.0	7.0	4.04	5.14
2	Placebo	27	5.22	0.93	17.9	3.0	5.0	5.0	6.0	7.0	4.85	5.59
	Laser	27	3.74	1.26	33.6	1.0	3.0	4.0	5.0	6.0	3.24	4.24
4	Placebo	27	2.81	0.96	34.2	1.0	2.0	3.0	4.0	5.0	2.43	3.20
	Laser	27	1.89	0.89	47.2	0.0	2.0	2.0	2.0	3.0	1.54	2.24
7	Placebo	27	1.59	0.93	58.4	0.0	1.0	2.0	2.0	3.0	1.22	1.96
	Laser	27	1.19	0.83	70.3	0.0	1.0	1.0	2.0	3.0	0.86	1.52

*SD* standard deviation, *CV* coefficient of variation (%), *Min* minimum, *Q1* 25^th^ percentile, *Med* median, *Q3* 75^th^ percentile, *Max* maximum, *CI* confidence interval for the mean

**Table 2:** Pairwise comparisons between laser and placebo-matched sides presented as mean pain difference in 27 patients (control pain minus experimental pain)

Day	Groups	N	Mean	SD	95 % CI		*P*
1	Control–laser	27	2.04	1.60	1.40	2.67	<0.0001
2	Control–laser	27	1.48	1.31	0.96	2.00	<0.0001
4	Control–laser	27	0.93	0.92	0.56	1.29	<0.0001
7	Control–laser	27	0.41	0.75	0.11	0.70	0.0088

*SD* standard deviation, *CI* confidence interval for the pain difference

### Pain changes over time

- *Control group*
  The one-way repeated-measures ANOVA showed a significant overall time-dependent decline in pain perceived in the placebo side (*P* < 0.0001). The Bonferroni test showed significant differences between each of the intervals (all *P* values ≤0.001).
- *Experimental group*
  The time-dependent pain decrease was significant in the laser side as well (ANOVA *P* < 0.0001). All pairwise comparisons were significant (all Bonferroni *P* values ≤0.005).

### Effect of treatment and time on pain

**Fig. 1:**
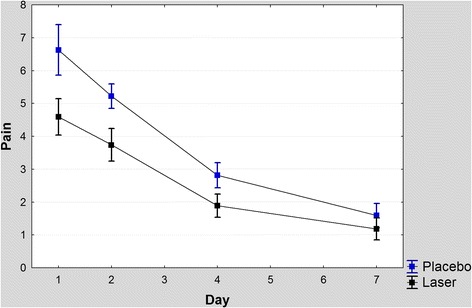
Pain levels on each side and at each evaluated day. *Error bars* represent 95 % confidence intervals

According to the two-way repeated-measures ANOVA, the effect of treatment (*P* < 0.0001) and time (*P* < 0.0001) were significant. The interaction of the variables “time and treatment” was not significant (*P* = 0.022). According to the Bonferroni post hoc test, all pairwise comparisons were significant (all *P* values <0.001, Fig. 1, Table 1).

## Discussion

Pain is a part of all orthodontic treatments [1, 3, 9, 14, 33], although its intensity, prevalence, and duration are disputed [1–7, 9–14, 16, 33–37]. About 90 % of patients experience pain during fixed orthodontic treatment [1–7, 11, 14]. In this study, all patients firstly felt pain in the first 24 h, which although decreased significantly, did not completely eliminate within 1 week. This was in line with earlier studies [1–3, 5, 6, 9, 16], most of which asserting that the pain peaks within the first 24 h and lasts for a short period [2, 5, 11–14, 33–35], while some others state that it might last for a rather long duration [6, 16]. Although not completely understood, orthodontic pain is mainly attributed to the compression of periodontal ligament under orthodontic forces [2, 7, 12, 14]. The immediate response to orthodontic forces characterizes by ischemia and PDL compression. After a few hours of prostaglandin release, the sensitivity of the pain receptors to noxious chemicals (e.g., histamine, bradykinin, acetylcholine, etc.) increases, marking the PDL hyperalgesia phase. This mechanism together with other phenomena (such as osteoclastic activity, neurogenic inflammation, and vasodilatation in the PDL) might cause pain [2, 3, 5, 7, 12, 14, 16, 35]. Different methods proposed to reduce orthodontic pain are NSAID consumption, chewing plastic wafers or gum, vibratory and transcutaneous electrical stimulation, and a diet of softer foods [7, 11, 15, 16]. It seems that fixed appliances might cause higher levels of pressure, tension, pain, and sensitivity of the teeth compared to removable appliances [13, 38]. However, the differences between the levels of pain treated with various fixed appliances such as with self-ligation, lingual, or conventional brackets were mostly not significant [33, 38, 39]. Recently, Invisalign approach has been suggested as a less painful method, although it has its own limitations [38].

Low-level laser therapy can be performed by He-Ne lasers. Irradiation with He-Ne laser at 632.8-nm wavelength and energy of 7.5 J/cm^2^ might reduce inflammation and accelerate the healing [40]. In this study, a single dose of He-Ne laser was shown effective in reducing the orthodontic pain sensed after beginning of tooth movement. There was no previous study on this particular type of laser, and all studies focused on laser wavelengths longer than ours. Therefore, we are limited to compare these results with other laser types. In this study, laser treatment contributed to about 12.1 % pain reduction in the laser side compared with the matched placebo side (1.21 out of 10 points). Our result was within the range reported in split-mouth studies [19, 26, 41] while it was smaller than the differences observed in parallel designs [18, 21, 42]. Of the few split-mouth studies conducted in this regard, only two found a significant difference. In one of them, laser irradiation accounted for 36.7 % pain reduction (3.67 out of 10) [26], while in the other one, laser reduced orthodontic pain for a statistically significant main score of 6.4 % (0.64 score out of 10) favoring laser irradiation [19]. The other two split-mouth designs failed to find a significant difference with very small differences (0.6 % in favor of the placebo side [41] and 2.4 % in favor of laser [17]). On the other hand, all parallel designs showed significant differences between the laser and placebo groups, with differences ranging from 19.6 to 52.5 % all favoring laser groups [18, 21, 42–44]. The differences can be attributable to the highly different methodologies including the orthodontic technique applied, laser dosimetry and parameters, the number of laser irradiation sessions, the laser types used, sample sizes, age ranges, gender compositions, analgesic consumption, and many other factors [15]. Mechanisms responsible for the pain-reducing effect of LLLT are unclear [15]. Perhaps, because of having anti-inflammatory and neural regenerative properties—as a probable result of photobioactive reaction which stimulates cell differentiation and proliferation—low-level laser therapy might be useful for pain control [20, 42–46]. Also, it might improve blood supply and enhance tissue recovery [42, 47]. Other factors contributing to the analgesic effect of LLLT might be the reactivation of enzymes targeted at pain-inductive factors, inhibiting nerve depolarization (C fibers in particular), ATP production, and prostaglandin reduction [15, 48]. Also, LLLT might alter nerve conduction by influencing the synthesis, release, and metabolism of encephalin and endorphins and many other neurochemicals [15, 49].

### Limitations and strengths

This study was limited by some factors. Pain is subjective, and numerous factors (such as sex, age, genetics, pain threshold, stress, emotional state, response to analgesics, sociocultural differences, past pain experiences, and the magnitude of the force applied) can affect it [1, 2, 4, 5, 7, 9, 11, 14–16, 30, 46, 32]. On the other hand, the sample size was based on a pilot study and the post hoc power was very high because of the specific design of the study, excluding the abovementioned confounding variables [30, 50]. Moreover, VAS is understandable by patients and is reliable, sensitive, and reproducible [5, 11, 12, 14, 16, 46]. Still, standardizing the intolerable pain was virtually impossible, as patients might have different levels of tolerance to pain. However, this could favor the generalizability since it was similar to what happens in a clinical condition, as what is relevant to patient is not a pain which can necessarily keep them awake at night (as might be incorrectly considered as a standardized response), but a pain which can render that specific patient seek emergency treatment.

Some studies did not exclude patients taking analgesics and only monitored the number of analgesics taken [19]. However, taking analgesics could disrupt the reliability and validity of the responses [30, 32]. Therefore, this and some other studies [21, 23] excluded such patients. Since there was no bias in delivering proper treatment towards the excluded patients and patients had voluntarily participated, they were unlikely secretly taking painkillers while falsely reporting the opposite. Therefore, the pain-related side effects might not be biased. It was possible that excluding patients consuming analgesics might skew the sample to more cooperating and psychologically prepared patients (and perhaps also to those with lower pains) [30]. However, including patients taking analgesics would not help in improving the generalizability, since they would as well perceive lower pains and skew the results [30]. Finally, the inclusion of both genders and a rather broad range of ages favored the generalizability, as pain perception might differ between ages [7, 16] and between genders [1, 3, 7]. The role of age in pain is debated, since the methodologies differ [3], and the correlation between pain threshold and age might be non-linear [7, 16]. There might be a linear negative correlation between general pain and age until the age 25 years [14, 16]. Nevertheless, in orthodontics, the relationship is not necessarily linear, and the most sensitive age might be between 13 and 16 years old [7, 14]. Some studies have observed more intense pains in older subjects [3, 14, 36] while some others have found no correlations between pain and age [12, 16, 33]. Besides sample and methodological differences, this again might be caused by a non-linear correlation pattern, with adolescence or another age range having lower pain thresholds compared to ages younger or older than it [7, 16]. With this in mind, enrolling subjects from different ages seem advantageous over pooling a narrow age range, since results of a study on pain in children might not be necessarily generalizable to pain perceived by adults and vice versa. Since, in this split-mouth design each subject was matched with himself/herself, such variations in patients’ demographics less likely confound the results, since the laser (treatment) sides were perfectly matched with their counterpart placebo quarters, in terms of age, gender, genetics, etc.

## Conclusions

Single-dose low-level laser therapy might reduce orthodontic pain caused by retracting maxillary canines. Regardless of the presence or absence of laser therapy, orthodontic pain might considerably decrease after a week, although not completely eliminated in this period.
